# Understanding the Utility of Less Than Six-Month Prognosis Using Administrative Data Among U.S. Nursing Home Residents With Cancer

**DOI:** 10.1089/pmr.2023.0047

**Published:** 2024-03-28

**Authors:** Long Vu, Siran M. Koroukian, Sara L. Douglas, Hannah L. Fein, David F. Warner, Nicholas K. Schiltz, Jennifer Cullen, Cynthia Owusu, Martha Sajatovic, Johnie Rose, Richard Martin

**Affiliations:** ^1^ Department of Population and Quantitative Health Sciences, School of Medicine, Case Western Reserve University, Cleveland, Ohio, USA.; ^2^ Case Comprehensive Cancer Center, School of Medicine, Case Western Reserve University, Cleveland, Ohio, USA.; ^3^ Center for Community Health Integration, School of Medicine, Case Western Reserve University, Cleveland, Ohio, USA.; ^4^ Frances Payne Bolton School of Nursing, Case Western Reserve University, Cleveland, Ohio, USA.; ^5^ Department of Sociology, University of Alabama at Birmingham, Birmingham, Alabama, USA.; ^6^ Center for Family and Demographic Research, Bowling Green State University, Bowling Green, Ohio, USA.; ^7^ Department of Internal Medicine, University Hospitals Cleveland Medical Center, Cleveland, Ohio, USA.; ^8^ Neurological and Behavioral Outcomes Center, University Hospitals Cleveland Medical Center, Cleveland, Ohio, USA.; ^9^ Departments of Neurology and of Psychiatry, School of Medicine, Case Western Reserve University, Cleveland, Ohio, USA.; ^10^ The Breen School of Nursing and Health Professions, Ursuline College, Pepper Pike, Ohio, USA.

**Keywords:** cancer, end-of-life care, nursing home, prognostication

## Abstract

**Background:**

There is a dearth of studies evaluating the utility of reporting prognostication among nursing home (NH) residents with cancer.

**Objective:**

To study factors associated with documented less than six-month prognosis, and its relationship with end-of-life (EOL) care quality measures among residents with cancer.

**Methods:**

The Surveillance, Epidemiology, and End Results linked with Medicare, and the Minimum Data Set databases was used to identify 20,397 NH residents in the United States with breast, colorectal, lung, pancreatic, or prostate cancer who died between July 2016 and December 2018. Of these, 2205 residents (10.8%) were documented with less than six-month prognosis upon NH admission. Main outcomes were more than one hospitalization, more than one emergency department visit, and any intensive care unit admission within the last 30 days of life as aggressive EOL care markers, as well as admission to hospice, receipt of advance care planning and palliative care, and survival. Specificity and sensitivity of prognosis were assessed using six-month mortality as the outcome. Propensity score matching adjusted for selection biases, and logistic regression examined association.

**Results:**

Specificity and sensitivity of documented less than six-month prognosis for mortality were 94.2% and 13.7%, respectively. Residents with documented less than six-month prognosis had greater odds of being admitted to hospice than those without (adjusted odds ratio: 3.27, 95% confidence interval: 2.86–3.62), and lower odds to receive aggressive EOL care.

**Conclusion:**

In this cohort study, documented less than six-month prognosis was associated with less aggressive EOL care. Despite its high specificity, however, low sensitivity limits its utility to operationalize care on a larger population of residents with terminal illness.

## Introduction

In the United States, 60% of cancer diagnoses occur among adults aged ≥65 years, and an estimated 9% of nursing home (NH) residents are diagnosed with cancer.^1,2^ However, there is no consistent approach in addressing end-of-life (EOL) care needs in this frail and vulnerable population.^3^ One proposal is documenting prognosis in NH residents with terminal illness.^3^

With respect to hospice eligibility, terminal illness is defined as having a less than six months life expectancy, documented by an attending physician.^4^ Within the NH setting, this information is reported by the Minimum Data Set 3.0 (MDS), a federally mandated process of comprehensive clinical assessments for all NH residents. To our knowledge, however, there is a lack of literature regarding the reporting of prognostication in the MDS, which is one of the few resources containing these data outside of electronic health records. Thus, there is a need to understand its potential utility as a measure in guiding care for NH residents with terminal illness.

In this study, we used the linked Surveillance, Epidemiology, and End Results (SEER), Medicare, and the MDS databases to study the utility of documented less than six-month prognosis (hereafter referred to as less than six-month prognosis), and its association with EOL care quality measures among NH residents with cancer who died from 2016 to 2018.

Using claims data, we identified markers of aggressive EOL care within the last 30 days of life—more than one hospitalization, more than one emergency department (ED) visit, and any intensive care unit (ICU) admission—which are associated with increased symptom burden and little benefit to overall survival, as well as higher incidence among NH residents with cancer than their community-dwelling counterparts.^5–9^ We also identified the receipt of advance care planning (ACP) and palliative care (PC) services, as well as admission and entry time into hospice as additional measures.

We hypothesized that compared with NH residents without less than six-month prognosis, those with less than six-month prognosis would have lower rates of aggressive EOL care, higher rates of receiving ACP and PC services, and both earlier and greater overall admission rates into hospice.

## Methods

### Data sources

This study used data from the linked SEER, Medicare, and MDS database from January 1, 2016 to December 31, 2018. The SEER captures incident cancers from population-based cancer registries across the United States, and encompasses ∼48% of the U.S. population within its covered areas.^10^

The linked Medicare database contains all Medicare beneficiaries in the SEER, represented by the Medicare Beneficiary Enrollment File (MBSF). The MBSF includes enrollment indicators in Medicare Parts A, B, C, D, and dual enrollment in Medicare and Medicaid. Medicare claims files included with the linked database are the Medicare Provider, Analysis, and Review (MedPAR) file for all inpatient hospital and skilled nursing facility admissions, and standard files for outpatient institutional and noninstitutional care, hospice care, and durable medical equipment.

The MDS is part of a federally mandated process for clinical assessment of residents in all Medicare and Medicaid certified NHs.^11^ MDS assessments are required upon admission, discharge, and periodic intervals for longer-term stays. MDS data consist of detailed clinical assessments of a resident's medical history, mental and physical disposition, and care received while in the NH.

This study was approved by the Case Western Reserve University institutional review board.

### Study population

Our study population consisted of NH residents diagnosed with active female breast, colorectal, lung, pancreatic, or prostate cancer at EOL, aged ≥65 years at time of diagnosis, who were admitted to an NH, and died between July 1, 2016 and December 31, 2018. These cancer types were selected to ensure that cancers with varying case-fatality rates were included, and because they account for nearly half of all incident cancers in the United States.

The study starting date was chosen to correspond with implementation of newly billable Current Procedural Terminology codes for ACP services at the time. At least one comprehensive MDS assessment conducted within the first 30 days of NH admission was required to ensure that any prognostication was documented. Residents later reporting less than six-month prognosis outside of the first 30 days of NH admission were excluded to accurately report on baseline and clinical characteristics. Residents with incomplete data fields necessary for calculating their Cognitive Function Scale score were also excluded.

To ensure proper capture of medical history, we limited our study population to only those with continuous fee-for-service Medicare coverage in the six months before NH admission until death. To validate the presence of active cancer at EOL, NH residents were screened for at least one inpatient or two outpatient claims coded for cancer, as specified by Clinical Classifications Software codes.^12^ Our final analytic sample size consisted of 20,397 residents (see Supplementary Fig. S1 for full inclusion criteria).

### Variables of interest

#### Main exposure

Our main exposure was a documented prognosis of less than six months life expectancy from any MDS assessment conducted within the first 30 days of NH admission. This item is coded as item J1400 and indicates whether or not a resident has a condition that may result in a life expectancy of less than six months.^13^ Guidelines state that this item may only be coded as “yes” when there is physician documentation that an NH resident is terminally ill within their medical record and/or is receiving hospice services.^14^ This information is collected by an MDS coordinator or MDS nurse who would have access to the NH resident's medical record.

Coding for the prognosis question was looked at after taking the most recent data field entry from all MDS assessments conducted within the first 30 days of NH admission. Although earlier intake assessments may report prognosis as missing, a later assessment would provide a definitive response.

#### Outcome variables

Our main outcomes of interest were the following markers of aggressive EOL care within the last 30 days of life: more than one hospitalization, more than one ED visit, and any ICU admission. We looked at admission to hospice at any time, and the receipt of any claim for ACP or PC services before death as additional quality measures. All outcome variables were derived from Medicare claims data (see Supplementary Table S1 for codes). Survival after NH admission was also examined.

#### Other independent variables

The following sociodemographic variables were collected from the MDS upon NH admission: age, sex, marital status, geographical region as defined by the U.S. Census Bureau, rurality as defined by the U.S. Census Bureau's 2010 core-based statistical area designations, and whether the initial NH stay was covered by Medicare. Race and ethnicity were recoded from the SEER as Hispanic (all races), non-Hispanic White, non-Hispanic Black, non-Hispanic Asian, and non-Hispanic Other, following reporting guidelines.^15^

Dual enrollment in Medicare and Medicaid was captured from the MBSF. Exact date of death was extracted from MBSF or MedPAR records; if unavailable from either, date of death was then imputed to the middle of the month (15th) using death variables from the SEER.

Comorbid conditions in the six months before NH admission were captured from Medicare claims data by Elixhauser Comorbidity codes.^16^ Cognitive status was measured by the Cognitive Function Scale using MDS items within the first 30 days of NH admission,^17^ and required complete-case data of associated items for study inclusion. Functional limitation was assessed using MDS items reporting on activities of daily living (ADLs) within the first 30 days of NH admission to calculate their Functional Limitation Scale score.^18^ The missing indicator approach was utilized for ADL items with any missingness to keep all remaining observations in the study.

### Statistical analyses

Survival after NH admission was examined using a Kaplan–Meier estimator. An unadjusted model and stratified model by cancer type examined differences between those with and without less than six-month prognosis. Median survival times (MSTs) are presented with 95% confidence intervals (CIs).

A confusion matrix evaluated the performance of less than six-month prognosis as an indicator for mortality, with less than six-month prognosis as the predictor, and death within six months of NH admission as the outcome. Performance metrics were specificity, sensitivity, positive predictive value (PPV), and negative predictive value (NPV).

Propensity score (PS) matching was used to minimize selection bias between cohorts, through the “matchit” R package.^19^ Suitability of PS matching was assessed by fitting a PS model with our independent variables and Elixhauser comorbidities. After determining a region of PS overlap (Supplementary Fig. S2), all NH residents with less than six-month prognosis were matched 1:1 to controls using a nearest neighbor (NN) matching algorithm. Covariate balance after matching showed improvement across all variables (Supplementary Fig. S3).

A second match was conducted using a full matching algorithm, due to a number of dropped controls from 1:1 NN matching. Full matching differs in that excess controls are not discarded, and that all observations at minimum, receive one match each.^20^ Covariate balance after full matching showed similar improvement (Supplementary Fig. S4). Considering the insignificant improvement over 1:1 NN matching, we present results using the full matched sample as additional sensitivity analysis.

To examine the association between less than six-month prognosis and our outcomes, we developed both unmatched and match-adjusted logistic regression models. The “marginal effects” R package was used to the estimate the marginal treatment effect among the treated in our adjusted models.^21^ Odds ratios, unmatched and match-adjusted odds ratio (aOR), are presented with 95% CIs.

All data processing was conducted with SAS, version 9.4, and statistical analyses were conducted using R, version 4.1.1.

## Results

Our study population consisted of 20,397 NH residents (median [interquartile range {IQR}] age, 79 [73–86]; 52.9% male) with active female breast, colorectal, lung, pancreatic, or prostate cancer before NH admission (Table 1). Among the NH residents in our study population, 2205 (10.8%) had documentation of less than six-month prognosis at the time of NH admission. Distribution of race and ethnicity between those with and without less than six-month prognosis was comparable.

**Table 1.** Patient Demographics of Nursing Home Residents With Cancer Who Died During 2016–2018, Stratified by Documented Less Than Six-Month Prognosis

	Overall (***N*** = 20,397)	With documented less than six-month prognosis (***N*** = 2205)	Without documented less than six-month prognosis (***N*** = 18,192)
Age group (%)			
65–74	6272 (30.7)	791 (35.9)	5481 (30.1)
75–84	8122 (39.8)	793 (36.0)	7329 (40.3)
85+	6003 (29.4)	621 (28.2)	5382 (29.6)
Age (median [IQR])	79.00 [73.00, 86.00]	79.00 [72.00, 85.00]	79.00 [73.00, 86.00]
Sex = male (%)	10,798 (52.9)	1101 (49.9)	9697 (53.3)
Race and Hispanic ethnicity (%)			
Hispanic	1021 (5.0)	94 (4.3)	927 (5.1)
Non-Hispanic			
White	16,384 (80.3)	1804 (81.8)	14,580 (80.1)
Black	2047 (10.0)	171 (7.8)	1876 (10.3)
Asian	826 (4.0)	120 (5.4)	706 (3.9)
Other	119 (0.6)	16 (0.7)	103 (0.6)
Marital status (%)			
Married	8436 (41.4)	722 (32.7)	7714 (42.4)
Widowed	6163 (30.2)	704 (31.9)	5459 (30.0)
Divorced/separated	2076 (10.2)	303 (13.7)	1773 (9.7)
Never married	2335 (11.4)	298 (13.5)	2037 (11.2)
Unknown	1387 (6.8)	178 (8.1)	1209 (6.6)
Cancer type (%)			
Lung	6891 (33.8)	840 (38.1)	6051 (33.3)
Breast	3487 (17.1)	342 (15.5)	3145 (17.3)
Colon	3462 (17.0)	390 (17.7)	3072 (16.9)
Pancreatic	1281 (6.3)	178 (8.1)	1103 (6.1)
Prostate	5276 (25.9)	455 (20.6)	4821 (26.5)
U.S. Geographic Region (%)			
Midwest	1917 (9.4)	314 (14.2)	1603 (8.8)
Northeast	9242 (45.3)	803 (36.4)	8439 (46.4)
South	3259 (16.0)	364 (16.5)	2895 (15.9)
West	5979 (29.3)	724 (32.8)	5255 (28.9)
Nonrural area of residence^a^ (%)	18,144 (89.0)	1795 (81.4)	16,349 (89.9)
Medicare/Medicaid dual enrollment (%)	5593 (27.4)	881 (40.0)	4712 (25.9)
Medicare-covered NH stay^b^ (%)	18,338 (89.9)	1348 (61.1)	16,990 (93.4)
Median survival time after NH admission (months [95% CI])	3.35 [3.25–3.45]	1.84 [1.71–1.97]	3.65 [3.52–3.78]

^a^ Rurality defined by U.S. Census Bureau 2010 core-based statistical area designations.

^b^ Coverage from Medicare Part A skilled nursing benefit.

CI, confidence interval; IQR, interquartile range; NH, nursing home.

Residents with less than six-month prognosis resided in nonrural areas at lower rates, compared with those without (81.4% vs. 89.9%). A greater percentage of NH residents with less than six-month prognosis was dually enrolled in Medicare and Medicaid (40.0% vs. 25.9%), but fewer had their initial NH stay covered under Medicare Part A skilled nursing benefit (61.1% vs. 93.4%). Residents with less than six-month prognosis presented with higher rates of cognitive impairment, functional limitations, and comorbidities (Supplementary Table S2).

Across cancer types, NH residents diagnosed with higher case-fatality cancers (i.e., lung and pancreatic) had slightly higher rates of less than six-month prognosis than those with other cancers (Supplementary Table S3). We also found it notable that 70.8% of NH residents with lung cancer, and 74.4% of those with pancreatic cancer within our study population had died within six months of NH admission, but only 12.2% of those with lung cancer, and 13.9% of those with pancreatic cancer had less than six-month prognosis.

**FIG. 1.**
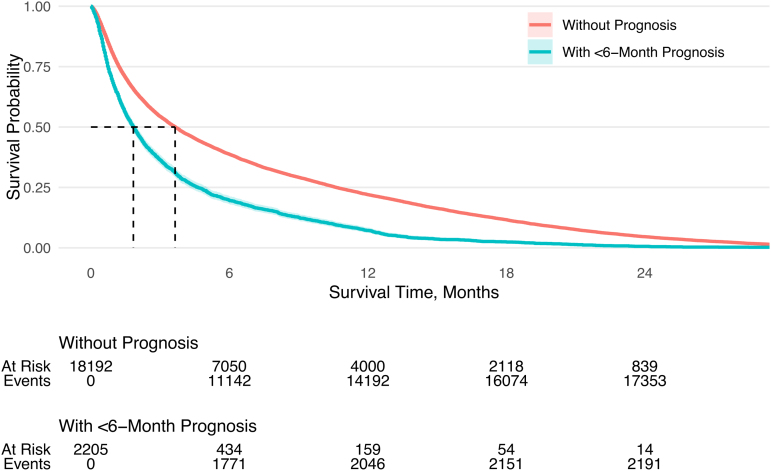
Kaplan–Meier survival curve of nursing home patients with cancer, stratified by documented less than six-month prognosis.

Unadjusted survival analysis showed that NH residents with less than six-month prognosis who died had an MST of 1.84 months (95% CI: 1.71–1.97) after NH admission, whereas those without had an MST of 3.65 months (95% CI: 3.52–3.78; Fig. 1). When stratified by cancer type, NH residents with lung or pancreatic cancer, and with less than six-month prognosis had the shortest MST at 1.51 months (95% CI: 1.38–1.68) and 1.28 months (95% CI: 1.02–1.58), respectively. Survival without less than six-month prognosis was 2.56 months (95% CI: 2.43–2.73) and 2.43 months (95% CI: 2.07–2.76), respectively (Supplementary Table S4).

Looking at the confusion matrix (Table 2), a high specificity of 94.2% was demonstrated, but sensitivity was low at only 13.7%. This indicated that although NH residents with less than six-month prognosis were indeed likely to die within six months after NH admission, a majority of cases were not captured. This is similarly reflected in the PPV and NPV, which were 80.3% and 38.8%, respectively.

**Table 2.** Confusion Matrix of Documented Less Than Six-Month Prognosis and Mortality Within Six Months of Nursing Home Admission

	Death within six months of NH admission (***N*** = 12,913)	Alive at six months after NH admission (***N*** = 7484)	Metrics	
With documented less than six-month prognosis (*N* = 2205)	1771	434	Sensitivity	0.137
Without documented less than six-month prognosis (*N* = 18,192)	11,142	7050	Specificity	0.942
			PPV	0.803
			NPV	0.388

NPV, negative predictive value; PPV, positive predictive value.

Across all markers of aggressive EOL care (Table 3), NH residents with less than six-month prognosis compared with those without had lower rates of more than one hospitalization (4.2% vs. 13.7%), more than one ED visit (11.1% vs. 21.7%), any ICU admission within the last 30 days of life (9.7% vs. 23.8%), and lower rates for receipt of ACP (17.3% vs. 22.1%) and PC services at any time (10.3% vs. 13.3%).

**Table 3.** Prevalence of End-of-Life Care Quality Measures Among Nursing Home Residents With Cancer Who Died During 2016–2018, Stratified By Documented Less Than Six-Month Prognosis

EOL care quality measures	Overall (***N*** = 20,397)	With documented less than six-month prognosis (***N*** = 2205)	Without documented less than six-month prognosis (***N*** = 18,192)
>1 Hospitalization in last 30 days of life	2590 (12.7)	92 (4.2)	2498 (13.7)
>1 ED visit in last 30 days of life	4185 (20.5)	244 (11.1)	3941 (21.7)
Any ICU admission in last 30 days of life	4537 (22.2)	213 (9.7)	4324 (23.8)
Admission to hospice at any time	11,249 (55.2)	1739 (78.9)	9510 (52.3)
Any claim for advance care planning	4410 (21.6)	381 (17.3)	4029 (22.1)
Any claim for palliative care services	2651 (13.0)	228 (10.3)	2423 (13.3)

ED, emergency department; EOL, end-of-life; ICU, intensive care unit.

A greater percentage of NH residents with less than six-month prognosis entered hospice at any time before death than those without (78.9% vs. 52.3%). Among those entering hospice, the median time from hospice entry to death for those with less than six-month prognosis was 40 days (IQR: 16–108), and 12 days (IQR: 3–46) for those without. Only 12.36% of NH residents with less than six-month prognosis had received hospice care for ≤7 days, in contrast to 40.45% without (Supplementary Fig. S5).

Residents with less than six-month prognosis had higher odds of entering hospice and lower odds of receiving aggressive EOL care across all measures in unmatched and match-adjusted models. Unmatched NH residents with less than six-month prognosis had 3.41 times the odds of entering hospice (95% CI: 3.07–3.79), 0.27 times the odds of more than one hospitalization (95% CI: 0.22–0.34), 0.45 times the odds of more than one ED visit (95% CI: 0.39–0.52), and 0.34 times the odds of any ICU admission within the last 30 days of life (95% CI: 0.29–0.39) when compared with those without. Match-adjusted models presented with comparable aORs with unmatched models (Table 4).

**Table 4.** Unmatched and 1:1 Nearest Neighbor Match-Adjusted Odds Ratios for Association of End-of-Life Care Quality Measures With Documented Less Than Six-Month Prognosis Versus Without

EOL care quality measures	Unmatched OR (95% CI)	1:1 NN match-adjusted aOR (95% CI)
>1 Hospitalization in last 30 days of life	0.27 (0.22–0.34)	0.34 (0.27–0.44)
>1 ED visit in last 30 days of life	0.45 (0.39–0.52)	0.51 (0.43–0.60)
Any ICU admission in last 30 days of life	0.34 (0.29–0.39)	0.49 (0.41–0.58)
Admission to hospice at any time	3.41 (3.07–3.79)	3.27 (2.86–3.72)
Any claim for advance care planning	0.73 (0.65–0.82)	0.86 (0.74–1.01)
Any claim for palliative care services	0.75 (0.65–0.86)	0.95 (0.78–1.15)

aOR, adjusted odds ratio; NN, nearest neighbor; OR, odds ratio.

For receipt of ACP and PC services, unmatched models initially showed NH residents with less than six-month prognosis having 0.73 (95% CI: 0.65–0.82) and 0.75 (95% CI: 0.65–0.86) times the odds than those without, respectively. After matching, however, receipt of ACP and PC services were no longer found significant (ACP aOR: 0.86 [95% CI: 0.74–1.01]; PC aOR: 0.95 [95% CI: 0.78–1.15]).

Sensitivity analysis conducted with the full matched sample yielded similar results (Supplementary Table S5).

## Discussion

In this population-based cohort study, we found that for NH residents with cancer, less than six-month prognosis was associated with significantly higher odds of hospice entry and lower odds of aggressive EOL care. Residents with less than six-month prognosis also enrolled in hospice earlier and thus for a longer duration, unlike many without less than six-month prognosis who enrolled in the last week before death. Prior studies show that those who spend longer time in hospice receive more appropriate symptom relief, less burdensome and higher quality EOL care, and greater concordance with EOL wishes.^22,23^

Although the overall documented rate of less than six-month prognosis was low at only 10.8% of the study population, this proportion aligns with other studies.^24–27^ The high specificity of less than six-month prognosis, and its association with lower aggressive EOL care speak to its potential utility. However, low sensitivity reduces broader application, likely stemming from low documentation. This is evident among those diagnosed with high case-fatality cancers, where lower rates of less than six-month prognosis did not align with expected mortality rates.^28^

This result also highlights a large group of NH residents with terminal illness who may be lacking access to EOL resources, despite efforts in recent years to improve PC accessibility and expand outside of just hospice benefit.^29–31^ If less than six-month prognosis indeed discourages aggressive EOL care, then improving documentation may represent an opportunity to address some of these care needs.

In another study using MDS 3.0 data, Thomas et al. developed the Mortality Risk Score (MRS3) from MDS items and compared against the Charlson Comorbidity Index and less than six-month prognosis from the MDS.^32^ They found the MRS3 was better at predicting 30- and 60-day mortality than both of these measures, and that less than six-month prognosis performed relatively poorly. Although this finding seemingly stands in contrast to our own, we note that their study population was much broader and not restricted to only NH residents with cancer, as well as differences in outcome measures.^32^ These differences in results also speak to the challenges with assigning prognosis itself, which is varied without consensus in approach.^33–36^

Although it is outside the scope of this study to investigate the financial factors influencing documentation of prognosis and related EOL care patterns, a sizable difference was observed between the number of NH residents with and without less than six-month prognosis who were dually enrolled in Medicare and Medicaid, as well as those who had a Medicare-covered stay under Part A skilled nursing benefit.^14^ Medicare Part A only fully covers the first 20 days of stay, and partially covers days 21 to 100 in an NH for those receiving skilled care,^37^ and hospice benefits require a terminal designation from care providers.^38^

### Limitations

These findings should be interpreted in light of the following limitations. First, this study relied on documentation from the MDS to define NH residents with less than six-month prognosis. Methodology used to determine their prognosis is unclear and fails to capture patients with rapid decline nearing EOL. Lack of reporting in the MDS also does not indicate survival believed to be greater than six months. Second, we were unable to ascertain whether a prognosis was explicitly disclosed to NH residents; and if so, whether if it would factor into their EOL care decisions.

Third, we lacked critical information on many factors that play a predominant role during the coordinated EOL decision-making process, such as rationale, presence of advance care directives, and influence of the family, among others. Fourth, our list of quality indicators was mainly process measures of care, and may not reflect the true NH resident experience in regard to care consistent with their wishes. Fifth, coding for utilization of ACP and PC services was relatively new during the time of our study window and, therefore, may be underreported.^39–41^

Lastly, usage of claims data necessitated restriction of our study population to only beneficiaries on continuous fee-for-service Medicare coverage. These results may not be generalizable to those enrolled in managed care, who were excluded, and represent a large and growing population of all Medicare beneficiaries.^42^

## Conclusions

We found that less than six-month prognosis was associated with lower odds of aggressive EOL care and greater odds of admission to hospice at any time, but was not associated with receipt of ACP or PC services for NH residents with cancer. Despite its high specificity, low sensitivity of less than six-month prognosis demonstrates a potential gap in access to EOL resources for a large number of NH residents with terminal illness. Our findings highlight the need to improve prognostic documentation within the MDS, as well as outlining a clear consistent approach in assigning prognosis to be used as an effective tool in identifying vulnerable residents nearing EOL and aid in guiding their care.

## Supplementary Material

Supplemental data

Supplemental data

Supplemental data

Supplemental data

Supplemental data

Supplemental data

Supplemental data

Supplemental data

Supplemental data

Supplemental data

## Abbreviations Used

ACP: advance care planning
ADL: activity of daily living
aOR: adjusted odds ratio
CI: confidence interval
ED: emergency department
EOL: end-of-life
ICU: intensive care unit
IQR: interquartile range
MBSF: Medicare Beneficiary Enrollment File
MDS: Minimum Data Set
MedPAR: Medicare Provider, Analysis, and Review
MRS: Mortality Risk Score
MST: median survival time
NH: nursing home
NN: nearest neighbor
NPV: negative predictive value
OR: odds ratio
PC: palliative care
PPV: positive predictive value
PS: propensity score
SEER: Surveillance, Epidemiology, and End Results
