# Supplementary material for: Understanding the Utility of Less Than Six-Month Prognosis Using Administrative Data Among U.S. Nursing Home Residents With Cancer
Source: Palliat Med Rep. 2024 Mar 28;5(1):127–35. doi: 10.1089/pmr.2023.0047 (PMC10979665; doi:10.1089/pmr.2023.0047)
Supplement: Supplemental data [file Suppl_TableS1.docx]

**Supplemental Table 1**. Codes used for identification of end-of-life care quality measures.

| **End-of-Life Care Quality Measures** | **Codes** |
| --- | --- |
| >1 hospitalization in last 30 days of life | Count of MedPAR records with different billing dates in last 30 days of life, excluding SNF stays |
| >1 ED admission in last 30 days of life | **Revenue center codes:**  "0450","0451","0452","0453","0454","0455","0456","0457","0459","0981"  **CPT/HCPCS:**  "99281","99282","99283","99284","99285" |
| Any ICU admission in last 30 days of life | **Revenue center codes:**  "0200","0201","0202","0203","0204","0207","0208","0209" |
| Admission to hospice at any time | Earliest available claim in hospice claims file with date of hospice start |
| Any claim for advanced care planning | **CPT/HCPCS:**  "99497","99498" |
| Any claim for palliative care services | **CPT/HCPCS:**  "99251","99252","99253","99254","99255","99341","99342","99343","99344",  "99345","99346","99347","99348","99349","99350" |

Abbreviations: emergency department (ED), intensive care unit (ICU)
