# Supplementary material for: Understanding the Utility of Less Than Six-Month Prognosis Using Administrative Data Among U.S. Nursing Home Residents With Cancer
Source: Palliat Med Rep. 2024 Mar 28;5(1):127–35. doi: 10.1089/pmr.2023.0047 (PMC10979665; doi:10.1089/pmr.2023.0047)

Supplemental Figure 2. Density plot of overlap in propensity scores between study cohorts of nursing home residents with documented <6-month prognosis and without.

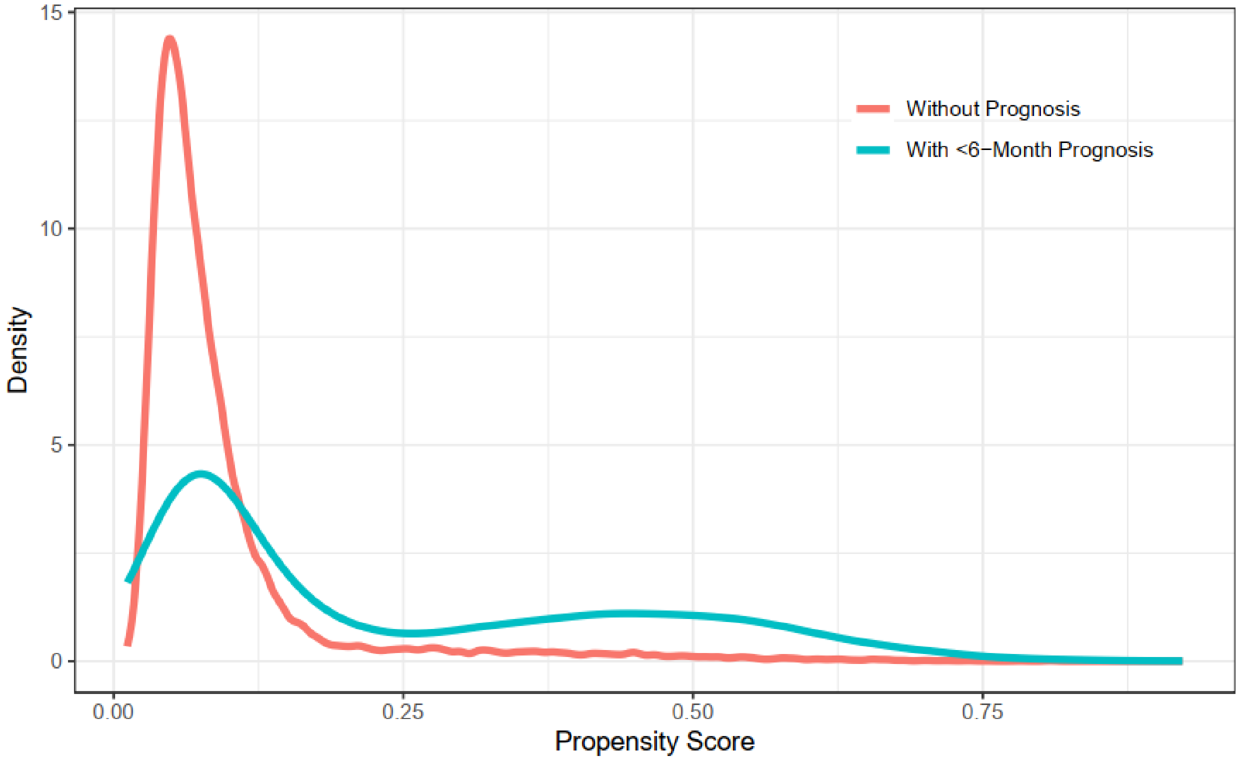

Supplement: Supplemental data [file Suppl_FigureS2.pdf]
