# Supplementary material for: Understanding the Utility of Less Than Six-Month Prognosis Using Administrative Data Among U.S. Nursing Home Residents With Cancer
Source: Palliat Med Rep. 2024 Mar 28;5(1):127–35. doi: 10.1089/pmr.2023.0047 (PMC10979665; doi:10.1089/pmr.2023.0047)
Supplement: Supplemental data [file Suppl_FigureS3.pdf]

Supplemental Figure 3. Love plot of covariate balance before and after 1:1 nearest neighbor matching.

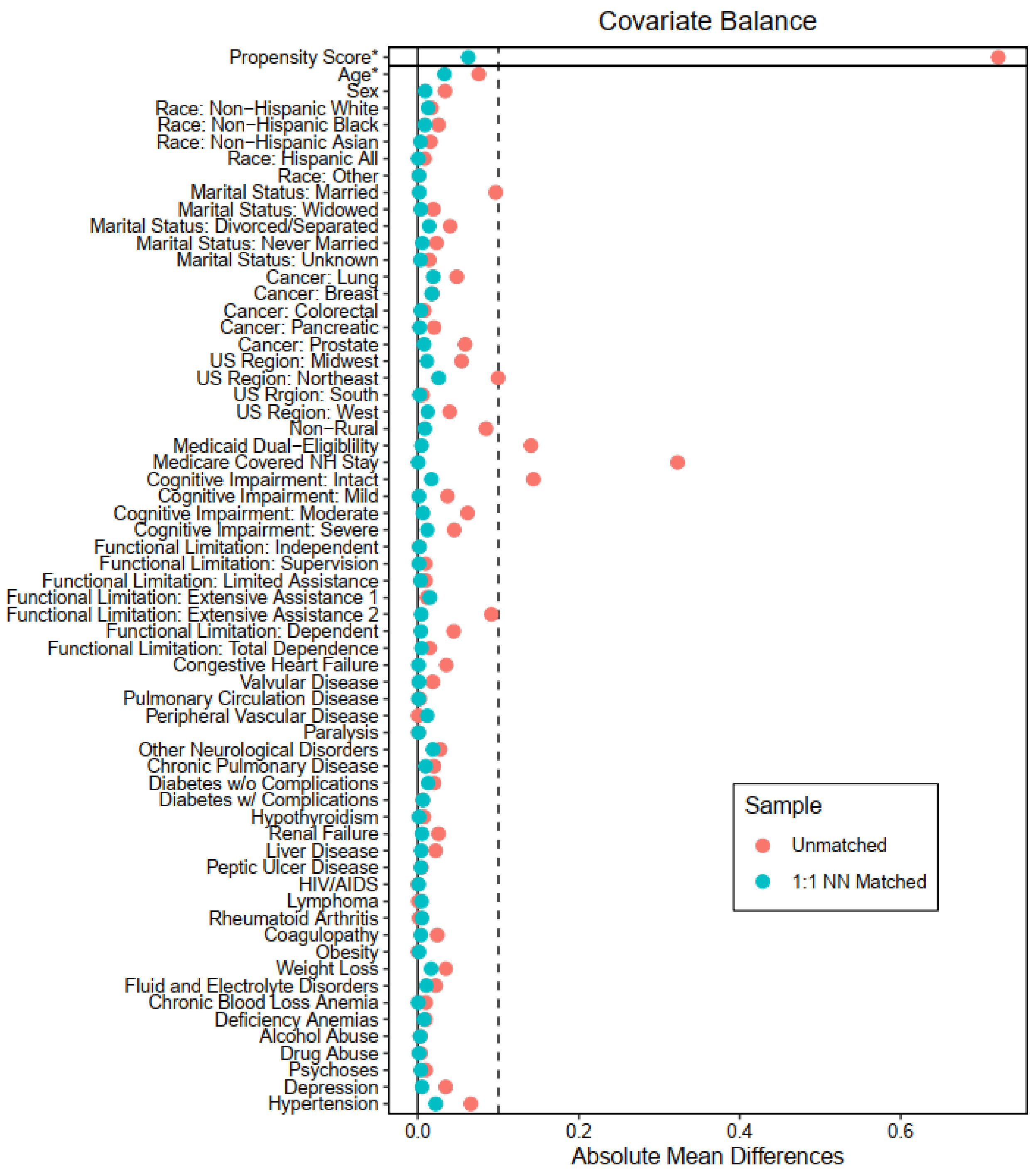

* indicates variable where absolute standardized mean difference is presented
