# Supplementary material for: Understanding the Utility of Less Than Six-Month Prognosis Using Administrative Data Among U.S. Nursing Home Residents With Cancer
Source: Palliat Med Rep. 2024 Mar 28;5(1):127–35. doi: 10.1089/pmr.2023.0047 (PMC10979665; doi:10.1089/pmr.2023.0047)
Supplement: Supplemental data [file Suppl_TableS2.docx]

**Supplemental Table 2**. Medical history of nursing home residents who died 2016-2018, stratified by documented <6-month prognosis.

|  | **Overall**  **N = 20,397** | **With Documented <6-Month Prognosis**  **N = 2,205** | **Without Documented <6-Month Prognosis**  **N = 18,192** |
| --- | --- | --- | --- |
| Cognitive Function Scale (%) |  |  |  |
| Cognitively Intact | 12,129 (59.5) | 1,029 (46.7) | 11,100 (61.0) |
| Mildly Impaired | 4,541 (22.3) | 563 (25.5) | 3,978 (21.9) |
| Moderately Impaired | 3,012 (14.8) | 447 (20.3) | 2,565 (14.1) |
| Severely Impaired | 715 (3.5) | 166 (7.5) | 549 (3.0) |
| Functional Limitation Scale (%) |  |  |  |
| Independent | 93 (0.5) | 14 (0.6) | 79 (0.4) |
| Supervision | 414 (2.0) | 63 (2.9) | 351 (1.9) |
| Limited assistance | 1,677 (8.2) | 200 (9.1) | 1,477 (8.1) |
| Extensive Assistance 1 | 3,071 (15.1) | 354 (16.1) | 2,717 (14.9) |
| Extensive Assistance 2 | 8,866 (43.5) | 779 (35.3) | 8,087 (44.5) |
| Dependent | 5,915 (29.0) | 727 (33.0) | 5,188 (28.5) |
| Total Dependence | 361 (1.8) | 68 (3.1) | 293 (1.6) |
| Elixhauser Comorbidities |  |  |  |
| Comorbidity Count (mean (SD)) | 3.78 (3.88) | 3.94 (3.40) | 3.76 (3.93) |
| Congestive Heart Failure | 4,435 (21.7) | 410 (18.6) | 4,025 (22.1) |
| Valvular Disease | 2,084 (10.2) | 189 (8.6) | 1,895 (10.4) |
| Pulmonary Circulation Disease | 1,227 (6.0) | 128 ( 5.8) | 1,099 (6.0) |
| Peripheral Vascular Disease | 2,950 (14.5) | 318 (14.4) | 2,632 (14.5) |
| Paralysis | 1,269 (6.2) | 139 (6.3) | 1,130 (6.2) |
| Other Neurological Disorders | 5,446 (26.7) | 643 (29.2) | 4,803 (26.4) |
| Chronic Pulmonary Disease | 5,317 (26.1) | 614 (27.8) | 4,703 (25.9) |
| Diabetes w/o Complications | 3,836 (18.8) | 454 (20.6) | 3,382 (18.6) |
| Diabetes w/ Complications | 3,057 (15.0) | 319 (14.5) | 2,738 (15.1) |
| Hypothyroidism | 2,481 (12.2) | 283 (12.8) | 2,198 (12.1) |
| Renal Failure | 3,621 (17.8) | 341 (15.5) | 3,280 (18.0) |
| Liver Disease | 1,339 ( 6.6) | 188 (8.5) | 1,151 (6.3) |
| Peptic Ulcer Disease | 486 (2.4) | 44 (2.0) | 442 (2.4) |
| HIV/AIDS | 32 (0.2) | < 11 | < 30 |
| Lymphoma | 503 (2.5) | 53 (2.4) | 450 (2.5) |
| Rheumatoid Arthritis | 533 (2.6) | 61 (2.8) | 472 (2.6) |
| Coagulopathy | 2,401 (11.8) | 212 (9.6) | 2,189 (12.0) |
| Obesity | 946 (4.6) | 103 (4.7) | 843 (4.6) |
| Weight Loss | 5,152 (25.3) | 625 (28.3) | 4,527 (24.9) |
| Fluid and Electrolyte Disorders | 8,339 (40.9) | 945 (42.9) | 7,394 (40.6) |
| Chronic Blood Loss Anemia | 878 ( 4.3) | 114 ( 5.2) | 764 ( 4.2) |
| Deficiency Anemias | 7,067 (34.6) | 783 (35.5) | 6,284 (34.5) |
| Alcohol Abuse | 309 (1.5) | 40 (1.8) | 269 (1.5) |
| Drug Abuse | 179 (0.9) | 26 (1.2) | 153 (0.8) |
| Psychoses | 525 (2.6) | 76 (3.4) | 449 (2.5) |
| Depression | 2,832 (13.9) | 374 (17.0) | 2,458 (13.5) |
| Hypertension | 9,933 (48.7) | 1,203 (54.6) | 8,730 (48.0) |
