# Supplementary material for: Understanding the Utility of Less Than Six-Month Prognosis Using Administrative Data Among U.S. Nursing Home Residents With Cancer
Source: Palliat Med Rep. 2024 Mar 28;5(1):127–35. doi: 10.1089/pmr.2023.0047 (PMC10979665; doi:10.1089/pmr.2023.0047)
Supplement: Supplemental data [file Suppl_TableS3.docx]

**Supplemental Table 3**. Distribution of documented <6-month prognosis among nursing home residents, stratified by cancer type.

| **Cancer Type** | **Total N** | **With Documented**  **<6-Month Prognosis (%)** |
| --- | --- | --- |
| Overall | 20397 | 2205 (10.8) |
| Lung | 6891 | 840 (12.2) |
| Breast | 3487 | 342 (9.8) |
| Colon | 3462 | 390 (11.3) |
| Pancreatic | 1281 | 178 (13.9) |
| Prostate | 5276 | 455 (8.6) |
