# Supplementary material for: Understanding the Utility of Less Than Six-Month Prognosis Using Administrative Data Among U.S. Nursing Home Residents With Cancer
Source: Palliat Med Rep. 2024 Mar 28;5(1):127–35. doi: 10.1089/pmr.2023.0047 (PMC10979665; doi:10.1089/pmr.2023.0047)
Supplement: Supplemental data [file Suppl_TableS4.docx]

**Supplemental Table 4**. Median survival times from Kaplan-Meier survival analysis, stratified by cancer type.

|  | **Median Survival Time, Months (95% CI)** | |
| --- | --- | --- |
| **Cancer Type** | **With Documented**  **<6-Month Prognosis** | **Without Documented <6-Month Prognosis** |
| Lung | 1.51 (1.38-1.68) | 2.56 (2.43-2.73) |
| Breast | 2.35 (2.35-2.99) | 4.70 (4.37-5.03) |
| Colorectal | 2.09 (1.81-2.53) | 4.64 (4.34-5.10) |
| Pancreatic | 1.28 (1.02-1.58) | 2.43 (2.07-2.76) |
| Prostate | 2.33 (2.07-2.89) | 4.57 (4.31-4.87) |

Abbreviations: confidence interval (CI)
