# Supplementary material for: Understanding the Utility of Less Than Six-Month Prognosis Using Administrative Data Among U.S. Nursing Home Residents With Cancer
Source: Palliat Med Rep. 2024 Mar 28;5(1):127–35. doi: 10.1089/pmr.2023.0047 (PMC10979665; doi:10.1089/pmr.2023.0047)

**Supplemental Figure 5.** Relative frequency histogram of time from hospice entry to death between nursing home residents with cancer, stratified by documented <6-month prognosis.

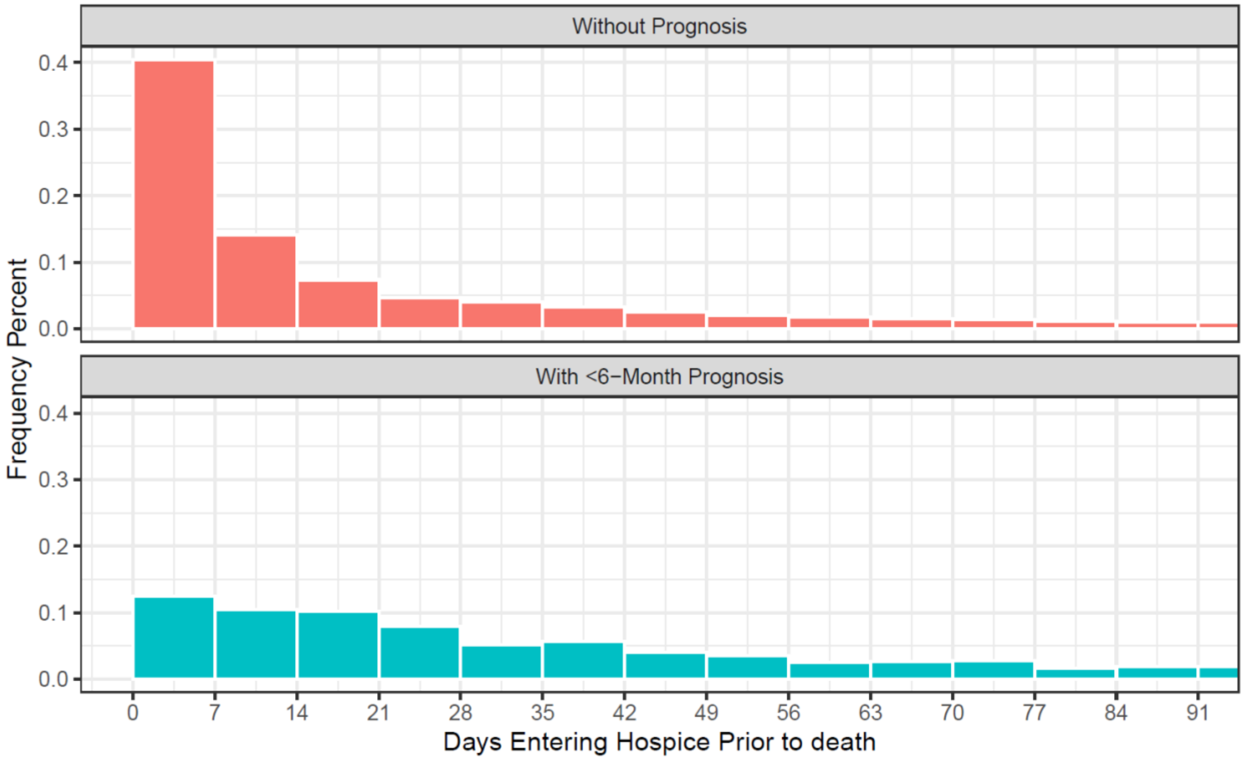

Supplement: Supplemental data [file Suppl_FigureS5.pdf]
