# Supplementary material for: Understanding the Utility of Less Than Six-Month Prognosis Using Administrative Data Among U.S. Nursing Home Residents With Cancer
Source: Palliat Med Rep. 2024 Mar 28;5(1):127–35. doi: 10.1089/pmr.2023.0047 (PMC10979665; doi:10.1089/pmr.2023.0047)
Supplement: Supplemental data [file Suppl_TableS5.docx]

**Supplemental Table 5.** Full match-adjusted odds ratios for association of end-of-life care quality measures with documented <6-month prognosis versus without.

| **End of Life Care Measures** | **Full Matched,**  **aOR (95% CI)** |
| --- | --- |
| >1 hospitalization in last 30 days of life | 0.36 (0.29-0.45) |
| >1 ED admission in last 30 days of life | 0.51 (0.44-0.61) |
| Any ICU admission in last 30 days of life | 0.50 (0.42-0.58) |
| Admission to hospice at any time | 3.43 (3.01-3.92) |
| Any claim for advanced care planning | 0.92 (0.80-1.07) |
| Any claim for palliative care | 0.91 (0.76-1.09) |

Abbreviations: match-adjusted odds ratio (aOR), confidence interval (CI), emergency department (ED), intensive care unit (ICU)
